# Integrative analysis of GEO data on the microbial community in colorectal cancer tissues and its impact on the tumor immune microenvironment

**DOI:** 10.3389/fonc.2026.1808851

**Published:** 2026-07-07

**Authors:** Bijun Zhou, Zhe Wang, Zhi Cao, Rong Liao, Wei Xiong

**Affiliations:** 1 Department of Clinical Medicine, Nanyang Medical College, Nanyang, Henan, China; 2 Department of Minimally Invasive Surgery, The Third Affiliated Hospital of Nanyang Medical College, Nanyang, Henan, China; 3 The First Department of Orthopedics, the Second Affiliated Hospital of Nanyang Medical College, Nanyang, Henan, China; 4 Department of General Surgery, the First Affiliated Hospital of Changsha Medical University, Changsha, Hunan, China; 5 Department of Medical, Hunan Traditional Chinese Medical College, Zhuzhou, Hunan, China

**Keywords:** colorectal cancer, GEO database, microbiome, multi-omics integration, tumor immune microenvironment

## Abstract

**Objective:**

Colorectal cancer (CRC) development is closely associated with gut microbiota dysbiosis and disruption of the tumor immune microenvironment. This study aimed to characterize and independently validate the cross-dataset associations of the “microbiome-immune axis” in CRC through multi-omics integration analysis and independent large-scale cohort validation methods.

**Methods:**

A multi-stage strategy was applied. The microbiome cohort GSE163366 was analyzed to identify gut dysbiosis in CRC. Single-cell transcriptomic data from GSE132465 characterized tumor immune remodeling. Cross-dataset association analysis linked microbial abundances (GSE163366) with immune cell proportions (GSE132465), and bulk transcriptomics explored underlying pathways. Independent validation cohort GSE237523 rigorously tested the robustness of core bacterial genera.

**Results:**

CRC patients exhibited significantly reduced α diversity (Shannon index, P = 1.29×10^-7^) and altered community structure (PERMANOVA, P = 0.001). The discovery set identified seven key differentially abundant genera, including pathogenic bacteria such as *Fusobacterium nucleatum* and *Bacteroides fragilis*, which were enriched in CRC, whereas beneficial bacteria such as *Akkermansia muciniphila* and *Roseburia intestinalis* were depleted. The tumor microenvironment exhibited features of reduced adaptive immune infiltration, including a significant reduction in B-cell proportion (11.5% decrease). Cross-dataset association analysis revealed that pathogenic bacteria showed positive associations with myeloid cells but negative associations with B-cells, whereas beneficial bacteria exhibited the opposite pattern. KEGG pathway enrichment analysis demonstrated that differentially expressed genes were significantly enriched in key immune signaling pathways, including the Toll-like receptor and NF-κB signaling pathways. Within the independent validation dataset GSE237523, all seven core bacterial genera identified in the discovery cohort were detected with significant abundance changes. Among these, six genera (85.7%) exhibited completely consistent directions of abundance change in the independent cohort. Notably, core pathogenic bacteria, including *Fusobacterium nucleatum* and *Bacteroides fragilis*, demonstrated high cross-cohort consistency.

**Conclusion:**

This study provides systematic characterization and independent validation of gut microbiota dysbiosis and its cross-dataset association with the immunosuppressive microenvironment of CRC. The reproducible abundance changes of six out of seven core microbial genera (85.7%) confirm cross-cohort robustness, offering a validated reference for understanding the microbiota-immune axis in tumor progression and developing microbiome-based diagnostic strategies.

## 1 Introduction

CRC ranks among the most prevalent malignant tumors globally, with high incidence and mortality rates, constituting a significant public health burden (1). Although its development is closely associated with genetic and epigenetic abnormalities, increasing evidence in recent years has highlighted the pivotal role of gut microbiota dysbiosis and disruption of the tumor immune microenvironment in the pathogenesis of CRC (2, 3). The gut, the largest microbial ecosystem in the human body, maintains a delicate dynamic equilibrium between its microbial community structure and the host immune system. Disruption of this balance, manifesting as dysbiosis, may drive the initiation and progression of colorectal cancer through multiple mechanisms (4).

Extensive research has demonstrated that the gut microbiota composition of patients with CRC differs significantly from that of healthy individuals, exhibiting a dysbiotic pattern characterized by the coexistence of enriched potential pathogenic bacteria and depleted beneficial commensal bacteria. Specifically, genera such as *Fusobacterium nucleatum* and *Bacteroides fragilis* are markedly enriched in tumor tissues. These bacteria promote tumor progression by activating carcinogenic signalling pathways (e.g., Wnt/β-catenin and NF-κB), inducing DNA damage, and recruiting immunosuppressive cells (5, 6). Concurrently, symbiotic bacteria with anti-inflammatory and barrier-maintaining functions, such as *Faecalibacterium prausnitzii* and *Roseburia intestinalis*, are markedly depleted (7). This microbial shift is intrinsically linked to remodelling of the tumor immune microenvironment. The immunological microenvironment of CRC often exhibits substantial heterogeneity. Some patients, particularly those with microsatellite instability-high (MSI-H), display an “immune-hot” phenotype and demonstrate favorable responses to immunotherapy. Conversely, a larger proportion of patients with microsatellite-stable (MSS) tumors present an immunosuppressive state characterized by impaired effector T-cell function and enrichment of regulatory T cells and M2 macrophages (8, 9). Gut microbiota and their metabolites, such as short-chain fatty acids and secondary bile acids, influence immune cell function through pattern recognition receptors (e.g., Toll-like receptors), thereby contributing to the shaping of this microenvironment (10, 11). Consequently, systematically elucidating the interactions between gut microbiota and the immune system in colorectal cancer is of considerable importance for advancing our understanding of its pathogenesis and identifying novel therapeutic targets.

Despite substantial advances in microbiome and tumor immunology research, the integration of these fields to systematically elucidate the role of the “microbiome-immune axis” in CRC remains limited. First, most studies are restricted to single-type data analyses, such as analyzing only 16S rRNA sequencing data or focusing exclusively on immune cell infiltration. There remains a lack of multi-omics integrated studies capable of simultaneously obtaining high-quality microbiome data, high-resolution immune cell atlases, and host whole transcriptome data from the same cohort of patient samples. This limitation hinders the identification of potential associations among microbes, immune cells, and host gene expression within the same patient population (12, 13). Second, the reproducibility and clinical translationtranslational potential of the existing findings are remain constrained. Many reported microbial biomarkers exhibit inconsistencies across different study cohorts, populations, and detection platforms, posing challenges for thereby limiting the clinical implementation of microbiome-based diagnostic or classification strategies (14, 15). Finally, there is still a lack of systematic-level elucidation and validation remain lacking regarding how gut microbiota regulate the functional states of specific immune cell subsets, such as B cells, myeloid cells, and mast cells, within the tumour microenvironment (16).

Through this “discovery-validation” analytical framework, the present study aimed to: (1) characterize CRC-associated dysbiosis and immune remodeling features in the discovery cohort; (2) construct and identify cross-dataset associations between microbial genera and immune cell profiles; (3) explore host molecular pathways potentially supporting these associations; and (4) validate the robustness of core microbial features in an independent cohort. By integrating multi-omics data with independent validation, this study provides a validated reference framework for understanding the role of the microbiota-immune axis in CRC pathogenesis.

## 2 Materials and methods

### 2.1 Data sources

Three datasets were downloaded from the GEO database (https://www.ncbi.nlm.nih.gov/geo/) for analysis. Microbiome data: Dataset GSE163366, comprising 16S rRNA gene sequencing data from 40 CRC tumor tissue samples and 40 normal colon tissue samples. Single-cell RNA sequencing data: Dataset GSE132465, comprising 10 normal colonic mucosal tissue samples and 23 CRC tumor tissue samples. Following data integration and quality control, 63, 689 cells were ultimately included in the analysis, including 16, 404 cells derived from normal tissue sources and 47, 285 cells derived from tumor tissue sources. The independent validation dataset GSE237523 comprised microbiome data from 114 CRC tissue samples and 114 normal colon tissue samples, totaling 228 samples. This dataset served as an independent cohort to validate the robustness of the key bacterial genera identified in the discovery cohort.

This study utilized publicly available human gene expression datasets deposited in the GEO database. All datasets were generated from previously published studies with appropriate ethical approvals obtained by the original investigators.

### 2.2 Microbiome data analysis

Raw sequence data were processed using the QIIME2 (v2022.11) workflow with Deblur-based quality filtering and Greengenes (v13.8) annotation. Data normalization was performed using a sum-of-relative-abundance transformation. Alpha and beta diversities (Bray-Curtis PCoA) were calculated using the vegan package in R (v4.3.0), with inter-group comparisons conducted using the Wilcoxon signed-rank test and PERMANOVA. Differentially abundant genera were identified using ANCOM-BC2 (v2.0.2; screening criteria: |log_2_FC| > 1, FDR < 0.05), and ALDEx2 (v1.30.0) was used for sensitivity analysis. Genera identified as significant by both methods were designated core taxa. For independent validation, the seven core genera were reanalyzed in GSE237523 using the Wilcoxon signed-rank test with the Benjamini-Hochberg correction, with an FDR < 0.05 considered statistically significant.

### 2.3 Immune cell analysis

Single-cell RNA-seq data underwent quality control, dimensionality reduction, clustering, and cell type annotation using the CellRanger and Seurat workflows. The CIBERSORT algorithm was used to quantify immune cell infiltration in bulk transcriptomic data from the GSE132465 dataset. Differences in immune cell proportions between the CRC and control groups were compared using the Wilcoxon signed-rank test.

### 2.4 Cross-dataset microbiome-immune association analysis

Based on the annotation results, proportional differences in the immune cell subpopulations between CRC and normal tissues were calculated using the GSE132465 dataset. Spearman’s rank correlation analysis (screening criteria: |r| > 0.3 and P < 0.05) was employed to investigate cross-dataset associations between microbial genus-level abundance (from GSE163366) and immune cell proportions (from GSE132465). Notably, these datasets were derived from independent patient cohorts; therefore, the correlation results represent cross-cohort associations rather than within-subject correlations. To assess the robustness of the resulting association network, sensitivity analyses were performed by varying the correlation threshold (|r| > 0.25, 0.30, and 0.35) and re-evaluating the network topology. Network structures that remained stable across the thresholds were considered robust. Adjustment for potential confounding variables (e.g., age, sex, and BMI) was not feasible because of incomplete clinical metadata in the public datasets; this limitation is addressed in the Discussion section. The igraph package was used to construct the association network, which was visualized and analyzed for topological properties using Cytoscape (v3.9.1).

### 2.5 Differential gene expression and pathway enrichment analysis

The limma package was used to analyze differentially expressed genes between CRC and normal tissues in the GSE132465 dataset (screening criteria: |log_2_FC| > 1 and adj.P.Val < 0.05). The clusterProfiler package was used to perform Gene Ontology (GO) enrichment analysis, including biological process (BP), cellular component (CC), and molecular function (MF), as well as Kyoto Encyclopedia of Genes and Genomes (KEGG) pathway enrichment analysis of the differentially expressed genes. The significance threshold for the enrichment results was set at p.adjust < 0.05.

### 2.6 Statistical methods

All statistical analyses were performed using R 4.3.0. Unless otherwise specified, hypothesis testing was conducted using two-tailed comparisons, with *P* < 0.05 considered statistically significant. Multiple comparisons were corrected for using the false discovery rate (FDR) method. Visualization was performed using R packages, including ggplot2 and pheatmap.

## 3 Results

### 3.1 Significant alterations in gut microbiota diversity and composition in colorectal cancer

**Figure 1:**
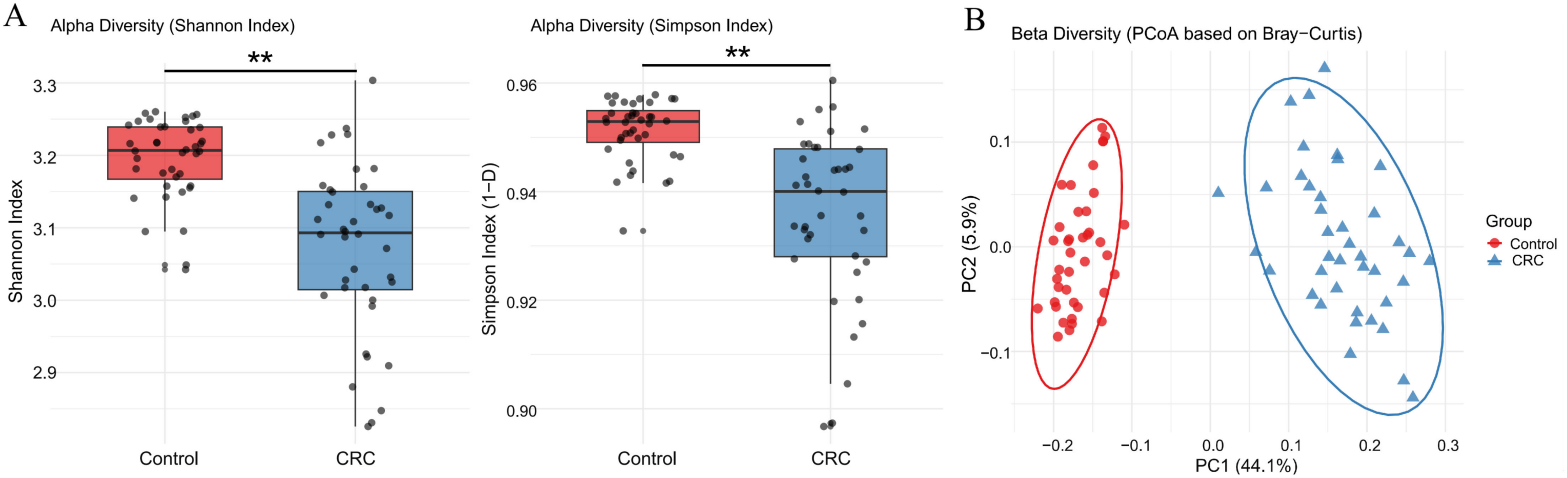
Comparison of gut microbiota diversity between CRC and control groups. **(A)** Box plots of α-diversity: left, Shannon index; right, Simpson index. **P < 0.001, Wilcoxon test. **(B)** PCoA analysis based on Bray-Curtis distance. Red dots represent the control group; blue triangles represent the CRC group.

Compared with healthy controls, α-diversity was significantly reduced in patients with CRC (Shannon index: CRC mean 3.072 vs. control 3.195, P = 1.29 × 10^-7^; Simpson index, P = 3.37 × 10^-7^) (**Figure 1A**). Beta-diversity analysis based on Bray-Curtis distance revealed significant community-level differences between groups (PERMANOVA R² = 0.412, P = 0.001), with PCoA demonstrating clear intergroup separation (**Figure 1B**).

**Figure 2:**
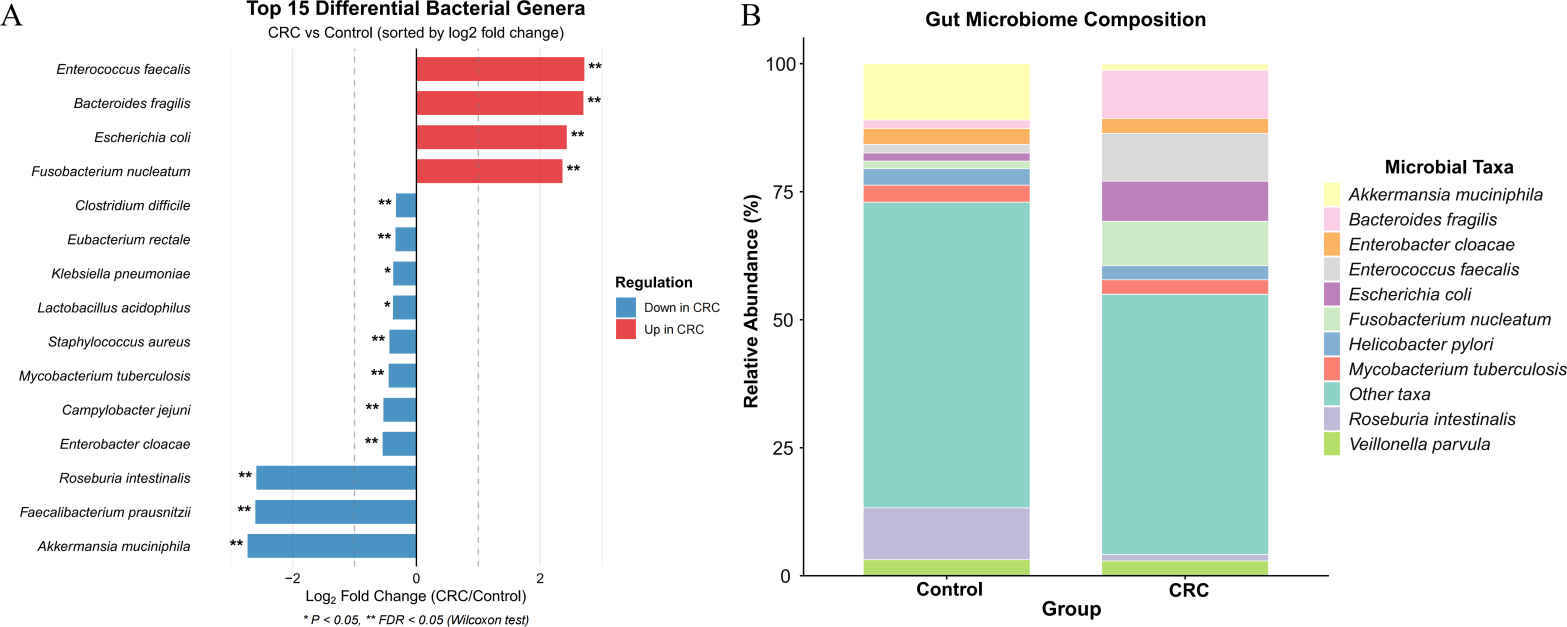
Gut microbiota compositional differences between CRC and control groups. **(A)** Exploratory differential abundance analysis showing the top 15 genera ranked by absolute log_2_FC value (DESeq2, FDR < 0.1). Red: increased in CRC; blue: decreased in CRC. **FDR < 0.05, *P < 0.05 (Wilcoxon test). Core genera validated by ANCOM-BC2 and ALDEx2 are reported in the main text. **(B)** Stacked bar plot of relative gut microbiota abundance (top 10 most abundant taxa). Depicts microbial composition in the control group (Control, n=40) versus the CRC group (n=40).

ANCOM-BC2 analysis identified seven core differentially abundant genera (|log_2_FC| > 1, FDR < 0.05). Genera enriched in CRC included *Fusobacterium nucleatum* (log_2_FC = 2.41), *Bacteroides fragilis* (log_2_FC = 2.55), and *Escherichia coli* (log_2_FC = 2.30), whereas genera depleted in CRC included *Akkermansia muciniphila* (log_2_FC = -2.63), *Roseburia intestinalis* (log_2_FC = -2.45), and *Faecalibacterium prausnitzii* (log_2_FC = -2.52). The ALDEx2 sensitivity analysis confirmed the directional consistency of six of the seven genera (**Figure 2A**). Relative abundance patterns demonstrated that *Bacteroides fragilis* (10.5%) and *Escherichia coli* (9.3%) were enriched in the CRC group, whereas *Akkermansia muciniphila* (7.4%) and *Roseburia intestinalis* (7.3%) were predominant in the control group (**Figure 2B**). These findings confirm pronounced dysbiosis in CRC, characterized by the enrichment of potential pathogenic bacteria and depletion of beneficial symbiotic bacteria.

### 3.2 The colorectal cancer tumour microenvironment exhibits coexisting immune suppression and innate immune activation

**Figure 3:**
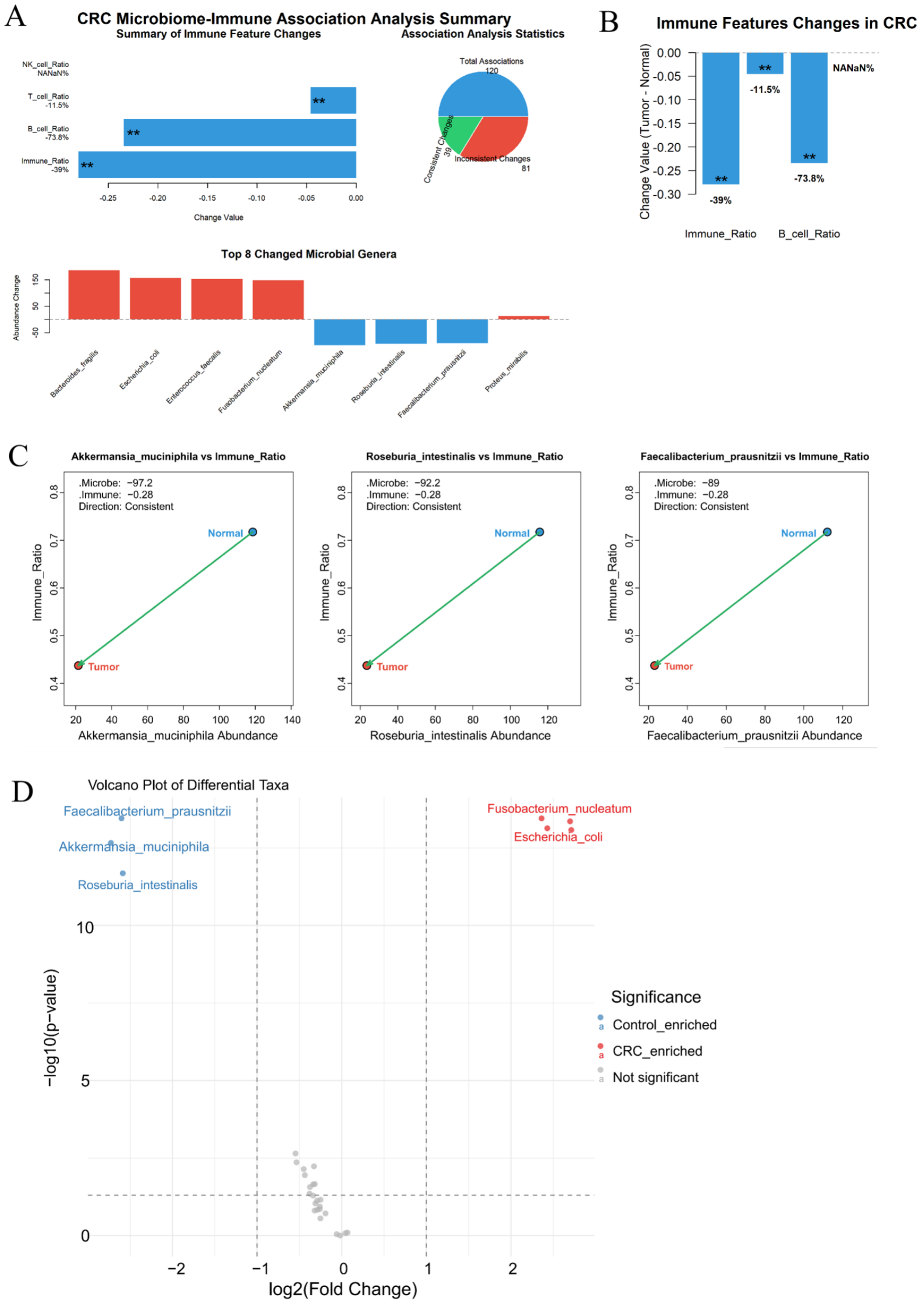
Immunological microenvironment characteristics of CRC tumours. **(A)** Comparison of immune cell proportions between colorectal cancer tissue and normal tissue. Bar charts illustrate differences in overall immune cell ratio (Immune_Ratio) and B-cell ratio between the colorectal cancer group and normal control group. Red bars represent normal tissue; blue bars represent CRC tissue. **P < 0.001, Wilcoxon test. **(B)** Quantitative bar chart of altered immune features in CRC. The x-axis denotes immune feature categories, including overall immune cell ratio, B-cell ratio, and other unlabelled features. The y-axis represents change values (Tumour - Normal), with negative values indicating decreased expression in CRC tissue. **(C)** Scatter plot correlating core beneficial bacteria with immune ratios. Demonstrates positive associations and synchronous decline trends between abundance of *Escherichia coli*, *Roseburia intestinalis*, and *Faecalibacterium prausnitzii* with immune ratios. Labels indicate abundance change magnitude (ΔMicrobe), immune ratio change magnitude (ΔImmune), and consistency of change. **(D)** Volcano plot of differential features between CRC and normal tissue, integrating microbial and immune cell characteristics. Red dots denote features upregulated in CRC, blue dots denote downregulated features.

Analysis of immune infiltration characteristics revealed systemic remodelling of the immune state within the CRC tumor microenvironment, exhibiting features of adaptive immune suppression coexisting with predominant localized innate immunity. Compared with normal tissue, the overall immune cell infiltration ratio in CRC tissue decreased by an average of 39%. Specifically, the B cell ratio decreased significantly by 11.5%, and the T cell ratio also decreased by 11.5%, whereas the NK cell ratio showed no significant change (**Figures 3A, B**). We note that the current analysis was limited to total B cell proportions; B cell subset composition was not resolved, and the functional implications of this reduction require further investigation.

Further analysis of the association between core microbiota and immune activity revealed that the abundance of beneficial bacteria, including *Akkermansia muciniphila*, *Roseburia intestinalis*, and *Faecalibacterium prausnitzii*, decreased by 97.2%, 92.2%, and 89.0%, respectively. Correspondingly, the overall immune ratio in these samples decreased by 0.28, exhibiting a consistent trend that suggests an association between the depletion of beneficial bacteria and reduced immune infiltration (**Figure 3C**). In contrast, certain innate immune cell populations exhibited increasing trends. The differential feature integration analysis identified nine significantly altered features. Innate immune cells, including mast and myeloid lineage cells, were markedly enriched in CRC tissues. It should be noted that “myeloid cells” represent an aggregate category; without further subtyping, this observation should be interpreted as indicating a general shift toward innate immune predominance rather than a specific mechanistic pathway (**Figure 3D**). This imbalance, characterized by reduced adaptive immune infiltration and localized innate immune predominance, may represent a feature that enable the CRC tumor microenvironment to evade immune surveillance and maintain a pro-tumor inflammatory state.

### 3.3 Cross-dataset microbial-immune associations and pathway enrichment analysis

**Figure 4:**
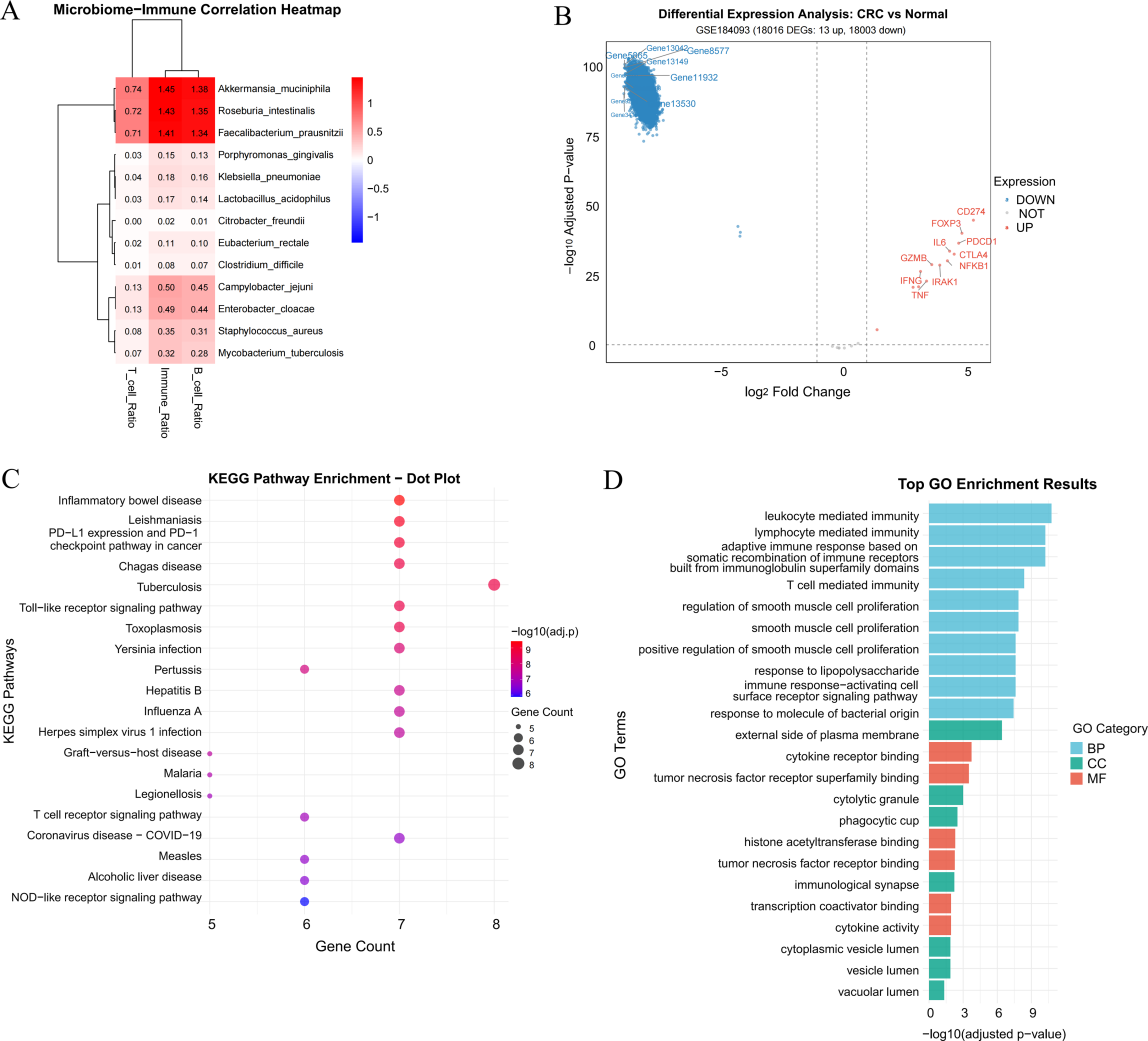
Cross-dataset microbial-immune associations and functional enrichment analysis. **(A)** Spearman correlation heatmap of core microbial genera and immune cell proportions. Red indicates positive cross-dataset association, blue indicates negative cross-dataset association. **(B)** Volcano plot of differentially expressed genes between colorectal cancer and normal colon tissues. X-axis: log_2_ fold change; Y-axis: −log_10_(P value). Each point represents a gene. Red dots indicate genes significantly upregulated in CRC; blue dots indicate significantly downregulated genes; grey dots indicate genes with no significant difference. **(C)** KEGG pathway enrichment dot plot for differentially expressed genes. Point size denotes gene count; colour intensity denotes −log_2_(p.adjust). The most significantly enriched pathways are displayed. **(D)** Bar chart of GO functional enrichment for differentially expressed genes. Three categories: Biological Process (BP), Cellular Component (CC), and Molecular Function (MF). X-axis, enrichment significance; Y-axis, significantly enriched GO terms, sorted by enrichment level.

To investigate the cross-dataset associations between gut microbiota composition and the tumor immune microenvironment, association analyses linking the abundance of microbial genera with immune cell proportions across independent cohorts were performed. Association heatmaps (**Figure 4A**) revealed that genera such as *Akkermansia muciniphila*, *Roseburia intestinalis*, and *Faecalibacterium prausnitzii* showed positive cross-dataset associations with overall immune activity and B cell proportion. Conversely, *Fusobacterium nucleatum* and *Escherichia coli* were positively associated with myeloid cells and negatively associated with B cells. These cross-cohort associations should be interpreted as hypothesis-generating observations rather than evidence of direct within-patient interaction.

Differential expression analysis identified 18, 016 differentially expressed genes (**Figure 4B**). KEGG pathway enrichment analysis (**Figure 4C**) revealed significant enrichment of these genes in Toll-like receptor signalling (p.adjust = 1.27×10^-20^), T-cell receptor signalling (p.adjust = 7.22×10^-17^), NF-κB signalling pathway (p.adjust = 3.60×10^-11^), and JAK-STAT signalling pathway (p.adjust = 5.49×10^-10^), among other immune regulatory pathways (17, 18). GO analysis (**Figure 4D**) further demonstrated that the differentially expressed genes were primarily involved in processes such as “leukocyte-mediated immunity” and “cytokine receptor binding”. The consistency between the association analysis and pathway enrichment results suggests that alterations in gut microbiota composition may be associated with widespread activation of host immune signalling pathways, thereby providing systematic clues for understanding the potential mechanisms through which microbiota influence the immunological microenvironment of CRC.

### 3.4 Systemic reconfiguration of the microbiome-immune association network in colorectal cancer

**Figure 5:**
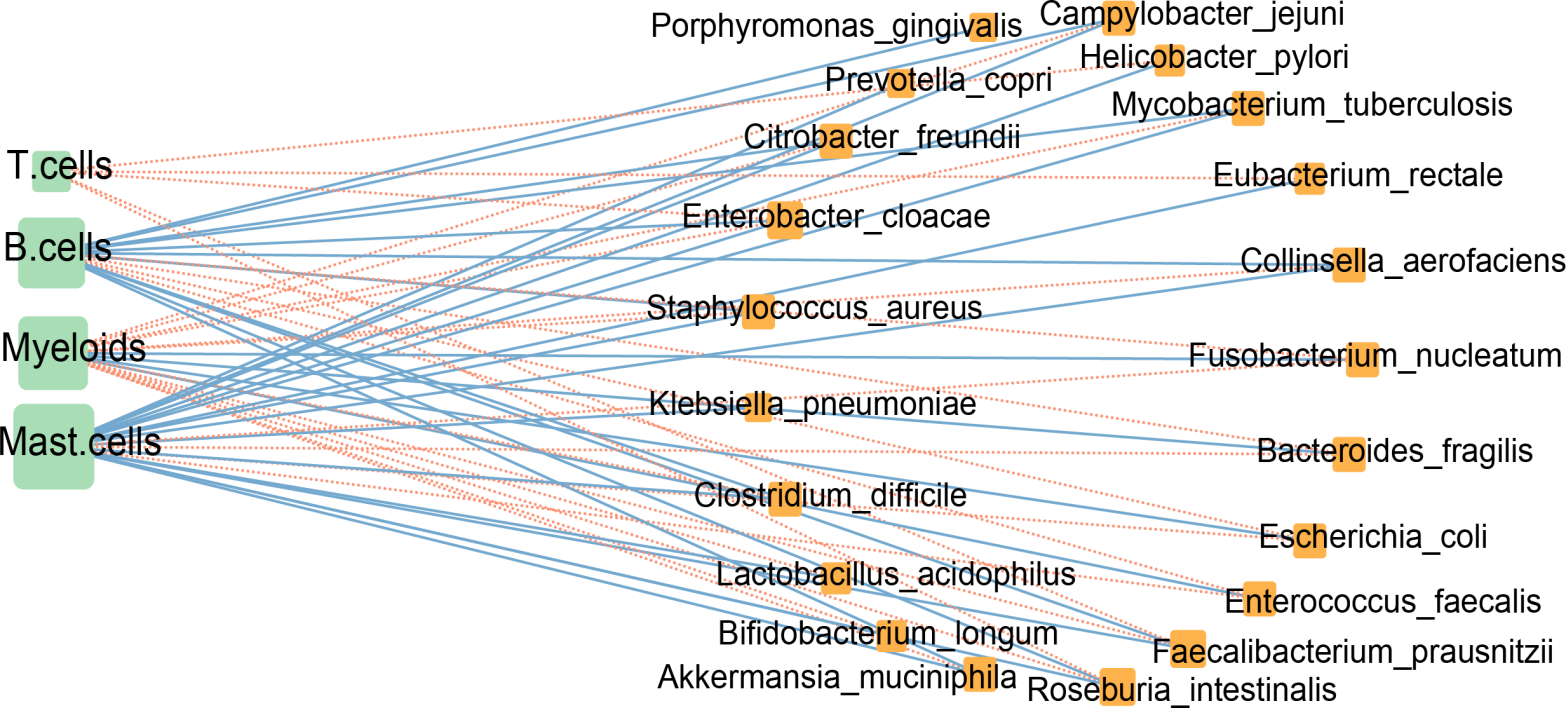
Cross-dataset association network between CRC-associated microbial genera and immune cell populations. The network comprises 34 nodes, including 30 microbial genera (orange) and four immune cell types (cyan: B cells, T cells, myeloid cells, and mast cells). Red dashed edges indicate positive cross-dataset correlations; blue solid edges indicate negative cross-dataset correlations (Spearman |r| > 0.3, *P <* 0.05). The node size reflects the degree of centrality. This network represents cross-cohort associations between independent microbiome and immune datasets, not within-subject interactions.

To investigate the cross-dataset associations between gut microbiota and immune cell profiles at the system level, a microbiome-immune association network was constructed (**Figure 5**). The network exhibited a distinct bipartite structure: a positively correlated module comprising potential pathogenic bacteria, represented by *Fusobacterium nucleatum* and *Escherichia coli*, together with myeloid cells, while beneficial bacteria, such as *Faecalibacterium prausnitzii* and *Akkermansia muciniphila*, formed another positively correlated module with B cells and mast cells. Network topology analysis revealed that mast cells occupied a central hub position, whereas certain pathogenic bacteria acted as pivotal “bridges” connecting distinct modules of co-expressed genes. Compared with normal tissue, the association network in CRC exhibited characteristics of connection sparsification and enhanced differentiation between modules, accompanied by a significant increase in the centrality of the pathogenic bacterial nodes.

Sensitivity analysis using different Spearman correlation thresholds (|r| = 0.25, 0.30, and 0.35) confirmed that this bipartite modular structure remained qualitatively stable, supporting the overall topological pattern. However, the strength of individual edges varied across thresholds. It is important to note that these associations represent cross-cohort correlations between independent microbiome and immune datasets rather than within-subject interactions and should, therefore, be interpreted as hypothesis-generating observations. These findings suggest that during CRC development, the coordinated relationship between the microbial community and host immune system may undergo substantial disruption and reconstruction.

### 3.5 Independent cohort validation of key bacterial genera

**Figure 6:**
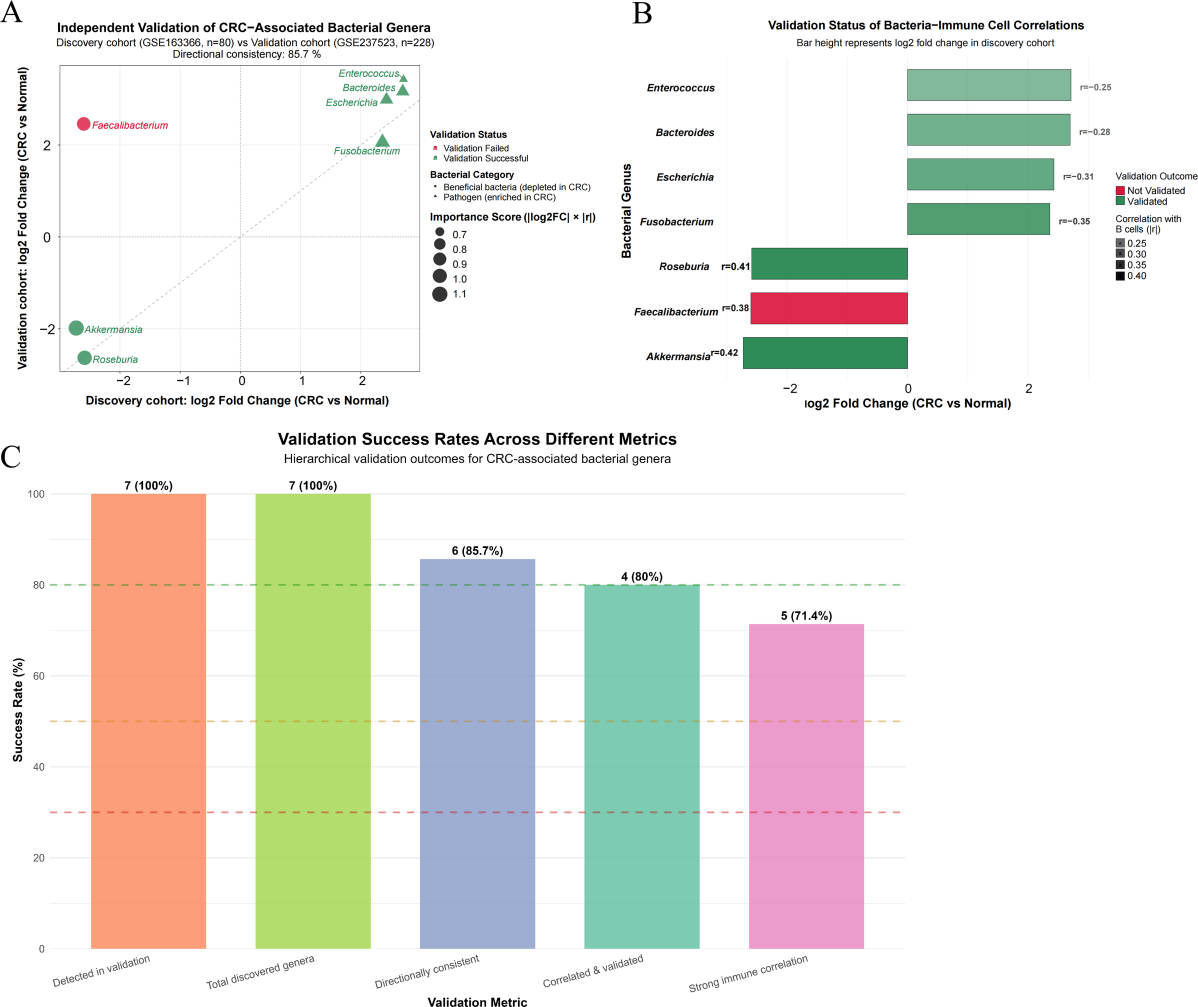
Independent cohort validation of core CRC-associated bacterial genera **(A)** Scatter plot comparing log_2_ fold change (log_2_FC) between discovery and validation cohorts. This plot illustrates log_2_ fold change values (CRC versus normal tissue) for seven key bacterial genera across the discovery cohort (GSE163366, n=80) and independent validation cohort (GSE237523, n=228). Triangles denote genera enriched in CRC (pathogenic bacteria), circles denote genera depleted in CRC (beneficial bacteria). Filled symbols indicate successful validation (consistent direction), hollow symbols indicate failed validation. The diagonal line represents perfect concordance. **(B)** Bar chart of validation of the immune-related genera status, displaying log_2_ fold change values in the discovery set and validation status. Bar transparency reflects absolute strength of B-cell association, with specific correlation coefficients (r) annotated above. Genera are sorted by log_2_ FC. **(C)** Stratified validation success rate statistics column chart, displaying success rates at each validation stage: total discovered genera, detection rate, directional consistency rate, proportion of strongly immunologically associated genera, and proportion of associated genera successfully validated. Dashed lines denote performance reference thresholds.

To ensure the reliability of the findings, we validated the seven key bacterial genera identified in the discovery cohort using an independent validation cohort, GSE237523 (n = 228). The results demonstrated that All genera were successfully detected and exhibited significant differences between the two groups. The direction of abundance change was reproduced in 85.7% (6/7) of the genera in the independent cohort (**Figure 6A**): *Fusobacterium nucleatum*, *Bacteroides fragilis*, *Escherichia coli*, and *Enterococcus faecalis* remained enriched in CRC, whereas *Akkermansia muciniphila* and *Roseburia intestinalis* remained persistently depleted. *Faecalibacterium prausnitzii* was the only genus to exhibit inconsistent validation results.

Further analysis revealed that most of the successfully validated genera, including *Fusobacterium nucleatum*, *Escherichia coli*, *Akkermansia muciniphila*, and *Roseburia intestinalis*, maintained strong correlations with B cells (|r| ≥ 0.3) (**Figure 6B**). Stratified validation rates demonstrated consistently high success rates from initial detection to final validation, confirming the robustness of the core genera across datasets (**Figure 6C**). This independent validation substantially enhances the credibility of our findings and provides important support for gut microbiota-based CRC biomarker studies.

## 4 Discussion

This study analyzed the cross-dataset associations between the gut microbiota and immune microenvironment in CRC by integrating microbiome, single-cell transcriptome, and bulk transcriptome data, alongside an independent validation cohort. The results confirmed a characteristic dysbiosis pattern in the gut microbiota of patients with CRC, featuring reduced α-diversity, enrichment of pathogenic bacteria, and depletion of beneficial bacteria, consistent with multiple previous studies (5–7, 13). The primary contribution of this study lies in validating the cross-cohort robustness of these core microbial features through an independent large-scale cohort (n = 228), rather than identifying novel bacterial taxa.

At the microbial community level, the observed genus-level alteration patterns closely aligned with the findings of numerous large-scale global CRC microbiome studies. For instance, the significant enrichment of F*usobacterium nucleatum* identified in this study is consistent with earlier findings by Kostic et al. and a global meta-analysis by Wirbel and Thomas et al., further confirming the pivotal role of this bacterium in CRC development (5, 13, 14). Similarly, increased abundance of *Bacteroides fragilis* and *Escherichia coli*, alongside reduced levels of *Akkermansia muciniphila* and *Roseburia intestinalis*, have been consistently reported across multiple independent cohorts (5, 7). These highly consistent patterns indicate that, despite population and geographical variations, the microbial characteristics associated with CRC exhibit substantial cross-population conservation.

The primary innovation and core finding of this study lies in independent cohort validation, confirming the high robustness of these core CRC-associated genera across different datasets. Validation using the GSE237523 dataset (n = 228) yielded an 85.7% directional consistency. Notably, similar highly reproducible bacterial features were observed in a meta-analysis by Wirbel et al., suggesting that the identified genera may constitute a core set of microbial markers for colorectal cancer (13).

Regarding the only inconsistently validated genus, *Faecalibacterium prausnitzii*, its opposite directional change (decrease in the discovery cohort but enrichment in the validation cohort) warrants further investigation. This inconsistency is not unprecedented; Thomas et al.’s multicohort study similarly reported heterogeneity in the abundance of this bacterium across populations (14). *Faecalibacterium prausnitzii* is one of the principal butyrate-producing genera, and its abundance is influenced by multiple factors, including host genetics and dietary fiber intake. This heterogeneity may reflect intrinsic differences among CRC subtypes or patient cohorts, suggesting that future microbial biomarker studies require more refined stratification analyses incorporating clinical and molecular subtyping (14, 19).

At the mechanistic level, this study identified differentially expressed genes significantly enriched in the Toll-like receptor (TLR) and NF-κB signalling pathways, consistent with established theories of microbiota-immune interactions. Pathogens such as *Fusobacterium nucleatum* can activate the NF-κB pathway through TLR4/MyD88 signalling, thereby promoting pro-inflammatory cytokine secretion, shaping an immunosuppressive microenvironment, and driving tumor progression (5). This mechanism is consistent with the findings of Dejea et al., who demonstrated that enterotoxin-producing *Bacteroides fragilis* promotes carcinogenesis through the activation of both NF-κB and Wnt pathways (20). Interestingly, this study also identified positive correlations between genes such as TLR3, TLR4, and IRAK1 and non-classically pathogenic bacteria, including *Methylibium* and *Sphingomonas*, consistent with the perspective proposed by Gopalakrishnan et al. that the gut microbiota broadly regulates immune responses through pattern recognition receptors (10).

The microbial-immune association network constructed in this study revealed that in CRC, network connectivity became sparser, modular differentiation increased, and the centrality of pathogenic bacterial nodes was elevated. This network restructuring aligns with the “tumour-associated inflammation” theory proposed by Grivennikov et al., whereby a chronic inflammatory microenvironment disrupts normal cell-cell and cell-microbe interaction networks, thereby creating conditions favorable for tumor progression (21). Notably, our study identified a significant reduction in the proportion of B cells within CRC tissues, which was negatively correlated with the enrichment of pathogenic bacteria. Although Sharonov et al. described the functional heterogeneity of B cells within the tumor microenvironment, the current data do not permit B cell subtyping. Therefore, the hypothesis that the observed reduction in B cells may reflect the selective loss of anti-tumor subsets remains speculative and requires dedicated investigation (16).

Several limitations of this study should be acknowledged. First, the microbiome data and single-cell transcriptomic data were derived from independent patient cohorts; therefore, the reported correlations represent cross-dataset associations rather than paired within-subject analyses and should be regarded as hypothesis-generating observations rather than evidence of direct causal relationships. Second, this retrospective analysis based on public databases could not adequately control for confounding factors such as medication use and diet; consequently, unmeasured variables may have influenced both microbial composition and immune infiltration, particularly within the microbiome-immune association network. Third, the immune characterization was relatively coarse. Myeloid cells were treated as an aggregate category without distinguishing functional subpopulations, and B-cell analysis was limited to total proportions without resolving subsets; therefore, the observed trends represent general immune landscape features rather than specific cellular mechanisms. Fourth, the gut microbiota-immune correlations identified in this study require further experimental validation to establish causality. Fifth, the analysis lacked stratification according to CRC molecular subtypes (e.g., MSI-H versus MSS) (9), which are known to influence both the immune microenvironment and microbial profiles. Future studies may integrate metabolomics, metagenomics, and spatial transcriptomics to dynamically monitor the evolution of microbial and immune networks during treatment in clinical cohorts. Such approaches may enable deeper exploration of the specific mechanisms through which microbial metabolites, such as short-chain fatty acids, modulate immune responses (11, 12).

In summary, through multi-omics integration and independent validation, this study not only reproduces key findings from previous research regarding microbial dysbiosis and immune suppression in CRC but also confirms the robustness of core microbial biomarkers through rigorous cross-cohort validation. These findings provide systematic evidence supporting the role of the “microbiota-immune axis” in CRC development, reinforcing the conservation of CRC-associated microbial characteristics and their potential clinical translational value. Furthermore, they establish an important theoretical and practical foundation for developing novel microbiome-based diagnostic and therapeutic strategies.

## 5 Conclusion

Through multi-omics integration and independent validation, this study systematically characterized the coordinated dysregulation of the microbiota-immune axis in colorectal cancer. The findings confirmed a characteristic gut microbiota pattern in patients with CRC, which demonstrated high cross-cohort reproducibility in an independent validation cohort (85.7% concordance for directional changes). The tumor microenvironment exhibits adaptive immune suppression, including reduced B cell levels, which show cross-dataset associations with specific microbiota alterations. Differentially expressed genes were significantly enriched in immune pathways, including Toll-like receptors and NF-κB, suggesting potential molecular links between microbiota dysbiosis and immune remodeling. This study offers a validated multi-omics reference framework for elucidating disease mechanisms and guiding the development of microbiome-based diagnostic strategies.

## Data Availability

The original contributions presented in the study are included in the article/supplementary material. Further inquiries can be directed to the corresponding author.
